# Crimean-Congo Hemorrhagic Fever Outbreak in Refugee Settlement during COVID-19 Pandemic, Uganda, April 2021

**DOI:** 10.3201/eid2811.220365

**Published:** 2022-11

**Authors:** Luke Nyakarahuka, Shannon Whitmer, Jackson Kyondo, Sophia Mulei, Caitlin M. Cossaboom, Carson T. Telford, Alex Tumusiime, Gloria Grace Akurut, Dianah Namanya, Kilama Kamugisha, Jimmy Baluku, Julius Lutwama, Stephen Balinandi, Trevor Shoemaker, John D. Klena

**Affiliations:** Makerere University, Kampala, Uganda (L. Nyakarahuka);; Uganda Virus Research Institute, Entebbe, Uganda (L. Nyakarahuka, J. Kyondo, S. Mulei, A. Tumusiime, J. Baluku, J. Lutwama, S. Balinandi);; Centers for Disease Control and Prevention, Atlanta, Georgia, USA (S. Whitmer, C.M. Cossaboom, C.T. Telford, T. Shoemaker, J.D. Klena);; Uganda Wildlife Authority, Kampala (G.G. Akurut, D. Namanya, K. Kamugisha)

**Keywords:** Crimean-Congo hemorrhagic fever, viral hemorrhagic fever, zoonotic, Bunyavirales, outbreak investigation, viruses, COVID-19, zoonoses, Uganda, coronavirus disease

## Abstract

Crimean-Congo hemorrhagic fever (CCHF) was detected in 2 refugees living in a refugee settlement in Kikuube district, Uganda. Investigations revealed a CCHF IgG seroprevalence of 71.3% (37/52) in goats within the refugee settlement. This finding highlights the need for a multisectoral approach to controlling CCHF in humans and animals in Uganda.

Crimean-Congo hemorrhagic fever (CCHF) is caused by CCHF virus (CCHFV). CCHF is a zoonotic disease that infects mainly livestock, and CCHFV is transmitted by ticks, primarily *Hyalomma* species. Humans are typically infected through contact with body fluids of infected livestock or through bites from infected ticks; human-to-human transmission of CCHFV has been documented (*1*), making early detection and infection prevention and control practices vital. The disease is distributed mainly in Central Asia, sub-Saharan Africa, and some parts of Europe (*2*). 

Uganda first reported a case of CCHF in 2013 in Agago district; subsequent sporadic cases have been detected throughout the country, especially within the cattle corridor (*3*–*6*). Serologic evidence indicates widespread infection among livestock in Uganda (*7*). We report the findings of epidemiologic and laboratory investigations conducted within a refugee settlement in the Albertine Graben region of Uganda after 2 human CCHF cases were confirmed during the COVID-19 pandemic and provide recommendations to reduce future transmission of CCHFV.

## The Study

**Figure 1:**
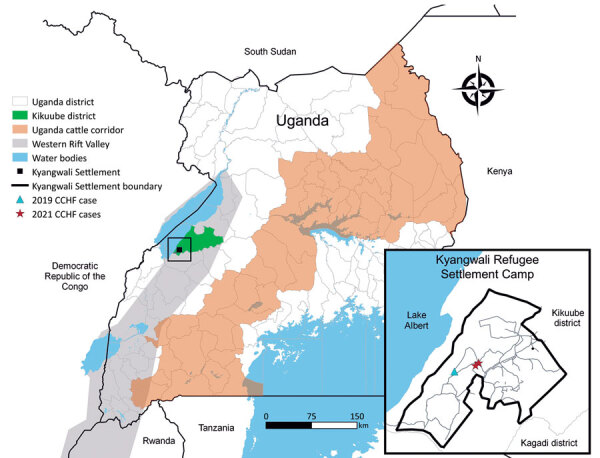
Location of Kyangwali Refugee Settlement (black box), Uganda, where 2 cases of Crimean-Congo hemorrhagic fever were reported during 2021. Inset shows close-up view of the settlement area, showing locations of 2021 cases and a previous case from 2019.

On April 27, 2021, a 16-year-old girl (patient 1) living in Kyangwali Refugee Settlement, Kikuube district, Uganda (Figure 1), sought care at a local health facility; clinical manifestations were a 2-day history of fever (38.1°C), headache, fatigue, hematemesis, and epistaxis. The patient had no history of recent travel outside the settlement and had stopped schooling because of national COVID-19 restrictions. The patient had been self-medicating for malaria without improvement; upon the onset of hemorrhagic signs, attending clinicians suspected a viral hemorrhagic fever because of the presence of epistaxis and hematemesis and implemented infection prevention and control measures, including patient isolation and use of personal protective equipment. A blood sample was collected and transported to Uganda Virus Research Institute the same day. Results revealed the patient was positive for CCHFV by real-time reverse transcription PCR (rRT-PCR) and had detectable CCHFV IgM and IgG on a US Centers for Disease Control and Prevention in-house ELISA specific to CCHFV (*3*).

On April 30, 2021, a 13-year-old boy (patient 2) from the same village was seen at the same health facility; symptoms were a 2-day history of fever that progressed into hemoptysis and epistaxis. The patient tested positive for malaria and received malaria treatment the day of symptom onset, but his condition did not improve. A blood sample was transported to Uganda Virus Research Institute and tested positive for CCHFV by rRT-PCR; CCHFV IgM and IgG were detected.

**Figure 2:**
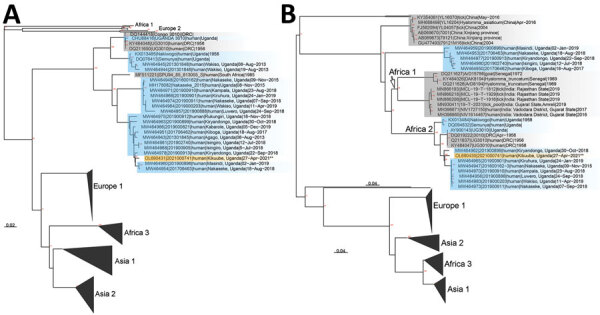
Phylogenetic analysis of all available full-length Crimean-Congo hemorrhagic fever (CCHF) small (A) and medium (B) segments from GenBank. Orange shading indicates sequence from 16-year-old girl in 2022; blue shading indicates past sequences from Uganda; gray shading indicates non-Uganda sequences. Major clades are labeled according to Balinandi et al. (*6*). Nodes with bootstrap support >70% are labeled in red. GenBank accession numbers are OL690430–1.

Both patients were managed clinically with supportive care, including intravenous fluids and paracetamol; they clinically recovered and were discharged. Next-generation sequencing analysis of nucleic acids extracted from a blood sample from patient 1 indicated the circulating virus grouped within the African 2 lineage, closely matching the virus from a case detected in the same area in 2019 (Figures 1, 2). Sequences were deposited into GenBank (accession nos. OL690430–1). Because of low viral load, generating a sequence from patient 2 was not possible.

Both CCHF patients were from the same village, attended the same church, and were refugees of Congolese origin; however, we did not identify any close contact or epidemiologic link suggestive of human-to-human transmission between them. Patient 1 had previously herded 15 goats that were housed in a barn adjacent to the family’s house and had not recently used tick control measures on the goats. Patient 2 lived near the goat pen of a neighbor in the camp. These goats, together with all livestock herds from the refugee settlement, grazed on communal land that was located <1 km away from the homes of the 2 confirmed patients, enabling easy mixing and transmission of pathogens.

We collected blood samples from 52 goats in the communal grazing land, including the 15 goats owned by patient 1’s family, and tested them by CCHFV rRT-PCR and CCHFV IgG ELISA. All 52 goats were rRT-PCR-negative, but 37/52 (71.3%) goats had detectable IgG. We also collected 14 ticks (*Rhipicephalus appendiculatus*) from the sampled goats; all tested negative for CCHFV by rRT-PCR.

## Conclusions

We describe 2 confirmed human CCHF cases in Kyangwali Refugee Settlement in Uganda. Both patients and their family members were rapidly identified and isolated upon seeking care at the health facility, and appropriate infection prevention and control measures were immediately implemented, which likely prevented onward CCHFV transmission. The presence of an isolation facility in the refugee settlement, set up as part of the COVID-19 response, played a key role in rapidly isolating patients. Both patients were given immediate supportive care and clinically recovered; human-to-human transmission of CCHFV was not identified.

In our investigation, 71.3% of sampled goats had detectable CCHFV IgG, indicating previous infection with CCHFV. As reported, the 2 patients were not living together and did not have direct contact with each other. These patients were likely infected through contact with infected body fluids of livestock or bites from infected ticks.

This investigation highlights some opportunities for improvement of viral hemorrhagic fever surveillance. Patient 1 self-treated with antimalarial medication before seeking care, which delayed isolation and increased the risk for CCHFV transmission, highlighting the importance of community education and encouraging health-promoting behaviors. Second, patient 2 initially tested positive for malaria, emphasizing the additional challenge in malaria-endemic countries of misdiagnosing viral infections such as CCHF as malaria. This challenge highlights the need for improved clinician awareness on the potential for malaria co-infections with other highly communicable pathogens such as CCHFV (G. Akurut, unpub. data); these infections cannot be differentiated without diagnostic testing. Improved diagnostic capability for multipathogen detection could also improve early identification and patient outcomes, as well as limiting the potential for transmission. 

Both patients sought care after developing hemorrhagic signs, which usually develop late in illness (*8*). This timing leads to delayed case detection, which could result in community transmission of the infection. Both patients had detectable CCHFV IgM and IgG, further supporting likely infection 7–10 days before diagnosis (*9*). Although healthcare workers suspected a viral hemorrhagic fever quickly on the basis of hemorrhagic signs, education is needed on early symptoms, which are nonspecific and can be confused with other causes of acute febrile illnesses, such as malaria or typhoid. Early suspicion and detection will reduce community and hospital transmission and improves the likelihood of early supportive care for and recovery of the infected persons.

Both patients were living in close proximity to goats; the livestock were located in the same compound for patient 1 and in a nearby goat pen for patient 2. Both patients reported close interaction with goats by way of grazing and tethering them. This close interaction with livestock increases the chance of zoonotic disease transmission. Livestock act as reservoirs for pathogens such as Rift Valley fever virus, CCHF, tuberculosis, and brucellosis and should be housed separately because they can act as carriers of infections to humans. The presence of ticks on the sampled goats demonstrates limited tick control in the herd. Tickborne diseases affect not only the health of humans but also livestock production. Vector control strategies coupled with improved management practices would improve these challenges, because communal grazing increases risk for pathogen transmission among animals from different herds.

This investigation of 2 confirmed CCHF cases highlights the rapid identification and intervention by healthcare workers and response from the Kikuube District health team and partners, such as the United Nations High Commission for Refugees, Prime Minister’s office, Medical Teams International, to successfully mitigate onward transmission of CCHFV in this vulnerable community. These efforts are particularly notable given the concurrent burden on the healthcare system and associated disease surveillance challenges from the COVID-19 pandemic.

## References

[1] Gürbüz Y, Sencan I, Oztürk B, Tütüncü E. A case of nosocomial transmission of Crimean-Congo hemorrhagic fever from patient to patient. Int J Infect Dis. 2009;13:e105–7. 10.1016/j.ijid.2008.08.002 18948048

[2] Messina JP, Pigott DM, Golding N, Duda KA, Brownstein JS, Weiss DJ, et al. The global distribution of Crimean-Congo hemorrhagic fever. Trans R Soc Trop Med Hyg. 2015;109:503–13. 10.1093/trstmh/trv050 26142451 PMC4501401

[3] Balinandi S, Patel K, Ojwang J, Kyondo J, Mulei S, Tumusiime A, et al. Investigation of an isolated case of human Crimean-Congo hemorrhagic fever in Central Uganda, 2015. Int J Infect Dis. 2018;68:88–93. 10.1016/j.ijid.2018.01.013 29382607 PMC5893389

[4] Kizito S, Okello PE, Kwesiga B, Nyakarahuka L, Balinandi S, Mulei S, et al. Notes from the field: Crimean-Congo hemorrhagic fever outbreak—Central Uganda, August–September 2017. MMWR Morb Mortal Wkly Rep. 2018;67:646–7. 10.15585/mmwr.mm6722a6 29879093 PMC5991810

[5] Mirembe BB, Musewa A, Kadobera D, Kisaakye E, Birungi D, Eurien D, et al. Sporadic outbreaks of crimean-congo haemorrhagic fever in Uganda, July 2018-January 2019. PLoS Negl Trop Dis. 2021;15:e0009213. 10.1371/journal.pntd.0009213 33684124 PMC7971858

[6] Balinandi S, Whitmer S, Mulei S, Nyakarahuka L, Tumusiime A, Kyondo J, et al. Clinical and molecular epidemiology of Crimean-Congo hemorrhagic fever in humans in Uganda, 2013–2019. Am J Trop Med Hyg. 2021;106:88–98. 10.4269/ajtmh.21-0685 34662872 PMC8733546

[7] Balinandi S, von Brömssen C, Tumusiime A, Kyondo J, Kwon H, Monteil VM, et al. Serological and molecular study of Crimean-Congo Hemorrhagic Fever Virus in cattle from selected districts in Uganda. J Virol Methods. 2021;290:114075. 10.1016/j.jviromet.2021.114075 33515661

[8] Paessler S, Walker DH. Pathogenesis of the viral hemorrhagic fevers. Annu Rev Pathol. 2013;8:411–40. 10.1146/annurev-pathol-020712-164041 23121052

[9] Shepherd AJ, Swanepoel R, Leman PA. Antibody response in Crimean-Congo hemorrhagic fever. Rev Infect Dis. 1989;11(Suppl 4):S801–6. 10.1093/clinids/11.Supplement_4.S801 2501854

